# Availability and affordability of priority lifesaving maternal health medicines in Addis Ababa, Ethiopia

**DOI:** 10.1186/s12913-022-07793-x

**Published:** 2022-04-20

**Authors:** Fantaye Teka Dinkashe, Kinfe Haile, Fatimetu Mohammed Adem

**Affiliations:** Department of Public Health, St. Paul’s Hospital Millennium Medical College, Addis Ababa, Ethiopia

**Keywords:** Maternal health medicine, Availability, Affordability, Addis Ababa

## Abstract

**Background:**

Access to life-saving medicines for maternal health remains a major challenge in numerous developing nations. Periodic and continuous assessment of access to lifesaving commodities is of enormous importance to measure progress and ensure sustainable supply. This study aimed to assess the availability and affordability of priority lifesaving maternal medicines in Addis Ababa in January 2021.

**Methods:**

An institutional-based cross-sectional study design was employed to assess 33 representative private pharmacies, public health facilities, NGO and private hospitals providing maternal health care and dispensing medicines from January 12 to 27, 2021 in Addis Ababa, the capital city of Ethiopia. WHO and Health Action International procedures were followed to determine sample size, sampling of health facilities, and data collection. WHO and UNFPA priority lifesaving maternal health medicines included in the Ethiopia essential medicine list were included in the study. Data were cleaned and entered into SPSS version 25 for analysis.

**Result:**

The overall mean availability of maternal health medicines was fairly high, 59% (range 6%-94%), as per the WHO availability index. Among the four sectors, the private pharmacy had the lowest availability (40%), while the mean availability in private hospitals, public and NGO/mission sector facilities were 70%, 72% and 72% respectively. Medicines used only for the management of maternal health conditions had lower availability (47%) compared to commodities used for the broader indication (65%). Compared based on source, the average availability of maternal health medicines which could be sourced locally was (68%) higher than imported medicines (55%). Affordability was not an issue in the public sector, public facilities offered maternal health medicines at no cost to the client. On the other hand, the private hospitals dispensed only 13% of the medicines at affordable prices followed by the private pharmacies (17%) and NGO/Mission facilities (29%). Furthermore, key challenges to access maternal health medicines were frequent stockouts in the public sector and the high cost of medicines in the private sector.

**Conclusion:**

Even though it was below the recommended 80% availability, fairly high availability with variabilities across sectors was observed. Except in the public sector, maternal health medicines were unaffordable in Addis Ababa.

**Supplementary Information:**

The online version contains supplementary material available at 10.1186/s12913-022-07793-x.

## Background

Most maternal deaths are preventable or treatable with verified, cost-effective interventions for infectious diseases and maternal complications [1]. Improving access to medicine is a basic component of reinforcing maternal health programs and results. Making fundamental maternal health drugs accessible to each woman when she gives birth will save the lives of 1.4 million women within the next 10 years [2]. In Ethiopia, inability to pay for medicines prevented more than half (55%) of women of reproductive age from obtaining advice or treatment, including seeking care during pregnancy and delivery [3].

WHO defines access to medicine as to be available in adequate amounts, in appropriate dosage, and quality at an affordable price for individuals and communities. In addition, It includes access to essential medicines as a core building block of a health system. [4, 5]. In 2011, UNFPA, UNICEF and WHO propelled the worldwide list of Priority Medicines for Mothers and Children, and updated the list in 2012 [6, 7]. It’s advised that improving availability, affordability and quality of these commodities is critical to reduce maternal deaths [8].

Ethiopia's health-service delivery is structured in a three-tier system: primary, secondary, and tertiary health care levels. The primary level is the most accessible service-delivery point, where basic health care is provided and managed while the upper levels manage more complicated cases [9]. Besides the public sector, the health care system is also augmented by different levels of clinics and hospitals which are operated by private for profit and non-governmental organizations (NGOs) [10]. To enable the public health institutions provide quality assured services, the Ethiopia pharmaceutical supply agency (EPSA) supplies essential pharmaceuticals at affordable prices in a sustainable manner [11, 12]. On the other hand, the private health facilities and drug retail outlets are generally supplied by private importers and wholesalers. However, the pharmaceutical pricing situation is characterized by the absence of clear medicines pricing policy, high retail markups, and high variation in prices of medicines [10].

Though access to essential medicines is a component of the fulfilment of the right to the highest attainable standard of health, women in low and middle-income countries face hurdles frequently [13]. The main reasons for the problem of access are erratic supply of pharmaceuticals, especially in public health facilities and unaffordable prices of essential medicines for the poor [10, 13]. Nevertheless, a reliable supply system to ensure health needs can be realized by public–private-NGO partnerships in supply delivery, proper regulatory control and exploring various purchasing schemes [14].

Established experiences demonstrated that availability and affordability of medicine depend on the demand and supply side factors. For example, registration and wider indication were reported attributes to access maternal health medicines [15, 16]. In addition, the service provision sector, source of supply and effective registration system are additional contributing factors [17–19].

Since studies conducted on the availability and affordability of priority life-saving medicines can help pinpoint gaps for policy analysis and solution development in low and middle-income countries [20], this study aimed to assess the availability and affordability of priority lifesaving maternal medicines in medicine outlets of Addis Ababa in January 2021.

## Methods

### Study setting and period

The study was conducted in Addis Ababa, the capital city of Ethiopia. Addis has a mix of health facilities comprising: 103 public health centers, 11 public hospitals, 33 private hospitals, and 270 pharmacies [21, 22]. Among these, all the public health centers, 10 of the public hospitals, and 24 of the private hospitals provide maternal health (MH) care services [21]. The data were collected from January 12 to 27, 2021.

### Study design

Institutional-based cross-sectional study supplemented with qualitative assessment was employed to assess the availability and affordability of life-saving maternal health medicines and key challenges.

### Selection of health facilities

In Ethiopia, a health center is the primary level of the healthcare system which provides promotive, preventive, curative and rehabilitative outpatient care including basic laboratory and pharmacy services for emergency and delivery services while hospitals provide services that require diagnostic facilities and therapeutic interventions with a minimum of gynecology, obstetrics and emergency services in addition to the services provided by health centers [23, 24].

The WHO-HAI methodology was used to select a representative sample of facilities from private pharmacies, public health facilities, NGO facilities and private hospitals [13]. According to the methodology, five facilities per sector sufficiently represent facilities in each survey area. However, increasing the sample size above the minimum would increase the survey’s accuracy [13]. Hence, the sample size was augmented by about 50% to improve accuracy. To represent each level of service provision in the public sector, four hospitals and four health centers were selected randomly.

**Fig. 1:**
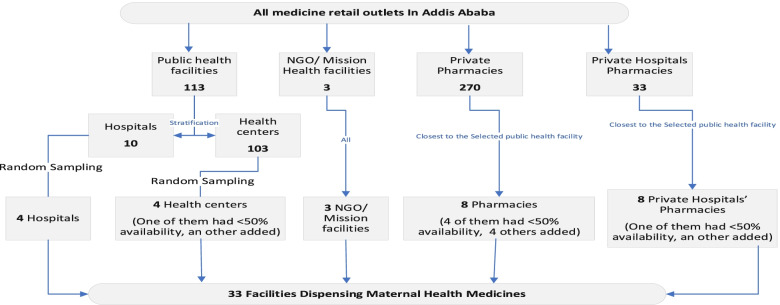
Health facilities Selection

Since one of the health centers had less than 50% availability, a health center from the backup list was included as per the methodology. Private pharmacies that were closest to each public facility were chosen. However, four of the primary selected private pharmacies had below 50% availability, and four additional private pharmacies were visited from the backup list. One additional private hospital was added from the backup for a facility below half percent availability. There were only three NGO or mission sector maternal health providers in Addis Ababa, and all of them were included in the study (Fig. 1).

### Selection of medicines

The commodities assessed were among Priority Medicines for Mothers which are used to manage major causes of maternal mortality as per recommendation by the UNFPA, UNICEF and WHO [25]. The WHO-HAI methodology medicine selection principles were considered for inclusion in the study. All of the medicines selected for this study were listed in the national essential medicine list of Ethiopia [26].The obstetrics protocol for health centers [27] and the obstetrics protocol of Ethiopia were reviewed to inform which medicines should be available at different levels of care [28]. The dosage of the medicines was sourced from the national obstetrics protocol, [28] the abortion care training manual [29], and sexually transmitted infections (STI) guidelines [30].

Medicines used for the management of postpartum hemorrhage: oxytocin, misoprostol, sodium chloride and ringer lactate; severe pre-eclampsia and eclampsia: magnesium sulphate, calcium gluconate, hydralazine and methyldopa; maternal sepsis: ampicillin, gentamicin and metronidazole; Safe/ Incomplete abortion and miscarriage: misoprostol and mifepristone; STI: azithromycin, cefixime and benzathine benzylpenicillin; preterm labour: nifedipine and dexamethasone; prevention of tetanus: tetanus vaccine were selected for the study [7]. Among them, oxytocin, magnesium sulphate, mifepristone and misoprostol, calcium gluconate and tetanus toxoid are considered as MH category while the rest of them are under broad indication.

### Operational definitions

#### Priority lifesaving MH medicines

Includes the WHO priority lifesaving medicines chosen according to 1) the global burden of disease; 2) the evidence of efficacy and safety for preventing or treating major causes of maternal mortality and morbidity [7].

#### Availability

Is readily accessible with ease, characterized by a resource that is committable, operable or usable upon demand to perform its required function [14]. A product is said to be available if it is available in the health facility providing MH service on the day of the visit. The following ranges were used for describing availability: < 30%—very low, 30% to 49% low, 50% to 80%—fairly high, > 80%—high [13].

#### Price

The amount the end-user paid to acquire the medicine during the data collection period.

#### Affordability

Selected courses of treatments that required more than 1 day’s wages for the lowest-paid unskilled government worker to purchase is considered unaffordable [13].

### Data collection tools and procedure

The WHO-HAI methodology and reliable data collection tool was used to capture required data from selected facilities. The method is rigorous, facilitating reliable data collection and valid analysis [13]. A semi-structured questionnaire was used to interview facility managers while ascertaining physical count of surveyed medicines using the checklist. To familiarize the data collectors with the tool, training was provided, and pilot tested in a public hospital and a private pharmacy, before the actual survey.

Trained data collectors visited medicine outlets in pair and recorded whether medicines were found, and their price in the recommended data collection checklist. For each medicine surveyed, data collectors recorded the stated product name of the lowest-priced generic medicines available, the manufacturer and the unit price of the product. In the public sector where medicines were free of charge to the care seekers, only availability was recorded. Data were collected on the same dosage form, and strength in all medicine outlets so that results are comparable [13].

The qualitative data were collected concurrently by interviewing the facility managers. The qualitative data were collected through face-to-face in-depth interviews. The questionnaire was semi-structured, and the data collector probed for key challenges and priority solutions to improve access to lifesaving maternal medicines. Each interview lasted an average of 15 min and was audio-recorded using a smartphone. A local language, Amharic, was used for interviewees' convenience. Data completeness, legibility and accuracy were supervised daily to ensure that all necessary data were collected properly. Field visits and follow-up telephone interviews were made to validate data in 4 of the sampled outlets. The number of registered suppliers for each product under investigation was collected from the Ethiopia Food and Drug Authority (EFDA) website (eRIS—Electronic Regulatory Information System (efda.gov.et)) [31].

### Data processing and analysis

Data were checked for completeness and consistency, and then entered into Statistical package for social sciences (SPSS) version 25. Descriptive statistics were used to summarize the findings and the results were presented as frequency tables and graphs. The availability of individual medicines was calculated as the percentage of sampled medicine outlets where the medicine was found. Data were reported in aggregate as public, private pharmacy, private hospital’s pharmacy or mission sector medicine outlets. WHO’s availability index was used to categorize the availability of the commodities from very low to high range [13].

The daily wage of lowest-paid government worker (LPGW) was used to determine the affordability of a full dose of each medicine or a total dose required for a monthly treatment. Excel based analysis tool was utilized for further processing of SPSS outputs. The lowest monthly salary scale was set at 1100 ETB giving the lowest daily wage to be 36.67 ETB (36.67 ETB = $0.92) according to the federal civil servants’ position rating, grading and salary scale, council of ministers’ regulation number 455/2019, [32, 33].

The qualitative data were analyzed using thematic analysis of the free text. The records were transcribed to the English language by the authors and verified by an expert in the college. During the thematic analysis, two coders, including the lead investigator and one expert with experience in qualitative data analysis coded the data to identify appropriate themes. Familiarization with the data; generating initial codes; searching for themes; reviewing themes; defining and naming themes were performed to identify key challenges and priorities to improve access to maternal medicines. The themes were described in narrative form, and later on, quoting the opinions of some respondents was added to the report.

## Results

### Availability of MH medicines

A total of 33 medicine outlets, which dispense priority lifesaving maternal health medicines were visited. The overall mean availability of maternal health commodities (MHCs) was fairly high landing at 59% (range 6%-94%), as shown below (Table 1). Private pharmacies had the lowest availability of MHCs (40%), while the availability in private hospitals, public and mission sector facilities was 70%, 72% and 72% respectively, depicting variability across sectors. In terms of individual medicine availability, Azithromycin 500 mg tablet had the highest availability (94%) followed by normal saline (88%) and ringer lactate (85%). On the other hand, misoprostol 200mcg tablet, benzathine penicillin and calcium gluconate were found in only 6%, 18% and 30% of facilities visited respectively.

**Table 1:** Percentage Availability of Maternal Health Medicines in Addis Ababa, January 2021

Maternal Health Medicines	Public Health Facility	Private Hospital's Pharmacy	NGO/ Mission Health Facility	Private Pharmacy	Total	WHO Availability Index
Ampicillin 1 g Injection	78%	67%	67%	67%	55%	Fairly High
Azithromycin 500 mg Capsule	78%	100%	100%	100%	94%	High
Sodium chloride 0.9% Isotonic in 1L Infusion	100%	100%	100%	67%	88%	High
Ringer lactate 1L Infusion	89%	100%	100%	67%	85%	High
Cefixime 400 mg Tablet	33%	100%	100%	75%	73%	Fairly High
Metronidazole 500 mg/100 ml Infusion	56%	100%	67%	58%	70%	Fairly High
Dexamethasone 4 mg/1 ml Injection	89%	67%	100%	50%	70%	Fairly High
Benzathine benzylpenicillin 2.4 million units in vial Injection	56%	0%	33%	0%	18%	Very Low
Gentamicin 40 mg/ml in 2 ml Injection	67%	67%	67%	17%	48%	Low
Magnesium sulfate 50%/10 ml Injection	89%	100%	100%	50%	79%	Fairly High
Methyldopa 250 mg Tablet	78%	33%	33%	67%	58%	Fairly High
Mifepristone + Misoprostol (200 mg + 200mcg) Tablet, Kit	78%	100%	67%	50%	73%	Fairly High
Nifedipine (Immediate) 20 mg Capsule	89%	67%	67%	42%	64%	Fairly High
Hydralazine 20 mg Injection	100%	67%	100%	0%	55%	Fairly High
Oxytocin 10 IU Injection	89%	67%	100%	0%	52%	Fairly High
TD (Tetanus, Diphtheria Toxoid) or TT (Tetanus Toxoid)	67%	67%	67%	0%	42%	Low
Calcium-gluconate 10%/10 ml Injection	33%	67%	33%	0%	30%	Low
Misoprostol 200 mcg Tablet	22%	0%	0%	0%	6%	Very Low
Average	72%	70%	72%	40%	59%	Fairly High

Generally, the maternal health commodities that had the highest availability (azithromycin, normal saline, and ringer lactate) are cross-cutting commodities that could be used in the management of multiple conditions. Of the medicines which are used only for maternal health conditions, magnesium sulphate (78%) and misoprostol + mifepristone kit (73%) had the highest overall availability. Oxytocin, which is used to induce labour and prevent post-partum hemorrhage, was available in about half of the surveyed facilities (52%) (Table 1).

The private hospitals were the leading to avail magnesium sulphate tablets, and misoprostol + mifepristone kit, which are used in the management of eclampsia/pre-eclampsia and safe abortion respectively. Misoprostol used to stop bleeding in post-partum hemorrhage was available only in public health facilities. Calcium gluconate used as an antidote for the management of toxicity of magnesium sulphate was available in one-third of public and NGO facilities, while none of the private pharmacies stocked it (Table 1).

Antibiotics as a pharmacologic class showed better availability. Azithromycin used for the management of uncomplicated genital chlamydial infections had the highest availability with 100% in the three sectors and 78% in the public sector. Benzathine penicillin used for the treatment of syphilis had the lowest availability among antibiotics; private hospital pharmacies were stocked out on the day of visit (0%). Gentamicin and ampicillin injections, used for the treatment of maternal sepsis, were available in about half of the facilities, though only these medicines were available at 17% of the private pharmacies (Table 1).

**Fig. 2:**
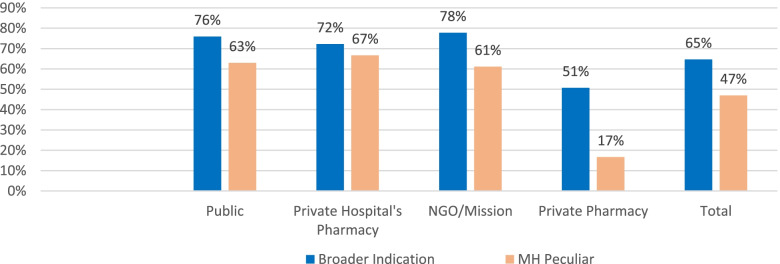
Availability of maternal health peculiar medicines compared with medicines for broader indication

Even though the sample size was limited, medicines used only for the management of maternal health conditions had lowest availability compared to commodities with broader indication, 47% and 65% respectively. Private pharmacy as a sector was found to have maximum difference for the category management, average availability of maternal health peculiar medicines was only 17% compared to the counter broader medicines (51%) (Fig. 2).

**Fig. 3:**
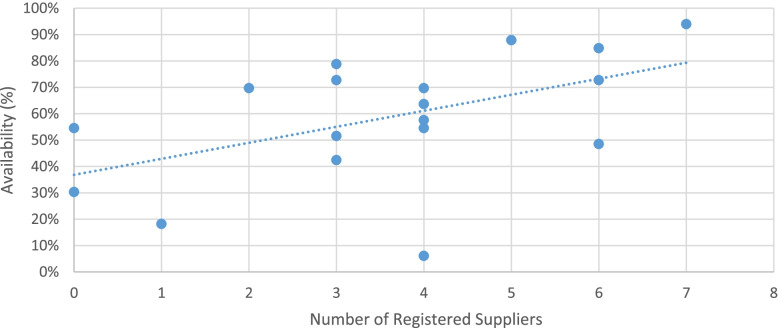
Availability of products with their number of in-country registered suppliers

Among the 18 maternal health medicines included in the study, only five were locally produced. The average availability of medicines that were produced locally was 68%, which is higher compared to imported medicines (55%). When the availability of a medicine was plotted with the number of registered suppliers, there appears to be an association between availability and the number of registered suppliers, where medicines with more registered suppliers had slightly higher availability (Fig. 3).

### Affordability of MH medicines

Public facilities in Addis Ababa offered maternal health medicines to clients at no cost, though there was variability in the implementation. One of the visited public health facilities had both free and paid maternal health medicines. The client has to pay for the medicines which were not included on the list of free medicines; these include dexamethasone injection, methyldopa tablet and nifedipine immediate-release tablet.

**Fig. 4:**
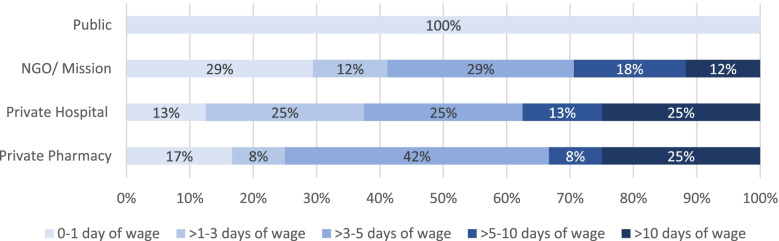
Days of wage required to cover the cost of maternal health medicines in each sector

Except in the public sector, maternal health medicines were not affordable. In the NGO and mission sector, 71% of maternal health medicines were found unaffordable, i.e., costing more than one day’s wage for a lowest-paid government employee. Private hospital pharmacies were the most expensive; only 13% of the medicines were found affordable and private pharmacies followed with 17%. In both private hospital pharmacies and private pharmacies, 25% of the medicines cost more than 10-day wages to cover the full treatment or a treatment for a month (Fig. 4).

Median day wages required to cover a full dose of acute treatment or monthly dose of maternal health peculiar medicines was 4 while it was 2.4 for medicines with broader indications; undeniably both were provided free of charge in the public sector. On the other hand, the number of registered suppliers appeared to have no trend with the affordability of treatments. In another comparison, maternal medicines which could be sourced locally costed lower median daily wages (1.3) than solely foreign-sourced medicines (2.9).

**Fig. 5:**
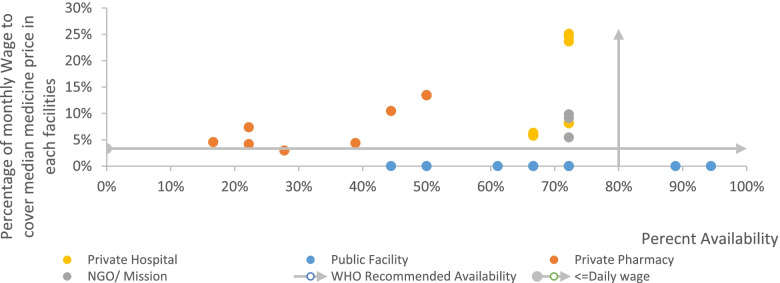
Median price and availability of maternal health medicines in health facilities in Addis Ababa

It is conceivable that LPGW cannot spend the whole of their daily income on medications. Nonetheless, the WHO- HAI methodology uses the daily wage of LPGW to determine the affordability of a course of treatment. To summarize our finding, taking the daily wage of an LPGW as a disposable income for medicine and 80% WHO recommended availability, only two public health facilities are in the desired quadrant (Fig. 5).

### Pharmacy managers’ interview

Pharmacy managers were asked about their perception regarding the key challenges to access priority maternal health medicines in their facility. Accordingly, the respondents first confirmed the existence of challenges in the supply management of lifesaving maternal health medicines. The interviews revealed various key challenges and identified priority solutions to improve access to priority lifesaving maternal health medicines. During the general analysis of the qualitative data, the responses towards the key challenges to access maternal health medicines fell under two themes: demand and supply themes.

Under the supply theme, participants mentioned frequent stock-outs, inadequate supply and cost of medicines as major challenges. In addition, public health facilities and private hospital pharmacies claimed the requested medicines were not supplied on time in sufficient quantity while private pharmacies and NGO/mission facilities experienced better refill rates. Public health facilities also complained about logistics issues for the supply of medicines while outlets that source from private sector suppliers were satisfied by the delivery of medicines to their store. A pharmacy head in one of the hospitals explained the problem as follows;

“We are expected to submit filled reporting and requestion format every two months as per the national integrated pharmaceuticals logistics system. The EPSA hubs are in charge of refilling our order within the next two weeks before our stock hits the emergency stock level. However, the refill is delayed, and the ordered quantity is cut by some amount or insufficient quantity to reach the maximum stock level is shipped to our warehouse. We dispense the stock in our store until it become zero and we might be stockout for a couple of days before the next shipment. Imagine what will happen for a poor mother visiting public facility for free service is forced to buy a medicine from a private pharmacy.” (Male pharmacy head, seven years of experience).

With the government initiative to promote health-seeking behavior and increase facility delivery, medicines and other supplies are provided for free in the public health facilities. But private sector providers claimed the cost of medicines for patients as a key challenge in their practice. One of the respondents elaborated as follows;

“We, the private pharmacies, are not receiving stock from the Ethiopian pharmaceuticals supply agency. We are deprioritized for their supply service. The private pharmaceuticals pricing in our country is not regulated at all! The private whole sellers decide the price of the medicine based on the product availability in the market and potential competition. If there is a shortage in the market it’s their time to boost their profit regardless of their cost to own the product.” (Female pharmacist, Four years of experience).

In the demand theme, some maternal health drugs, such as fluids and oxytocin injection, were not in high demand in private pharmacies. Because of the demand, the private pharmacies were not interested to stock the drugs to avoid expiry and wastage. A senior pharmacist in one of the private pharmacies described;

“Some of the medicines are prescribed exclusively for maternal health services like for the management of third stage of labour. Health facilities providing such services ensure commodity security by any means possible for such services due to their emergency nature. Unless there is unexpected stock out in the facility, all the prescriptions are filled by themselves. I don’t want to stock since the probability for expiry is high due to the demand.” (Male pharmacist, seven years of experience).

In all sectors, there was no unmet training demand for supply chain management of maternal health drugs and none of them noticed any client reluctance to access maternal health medicines. Responses on how to improve access to maternal health medicines were coded into three themes during the analysis. The three themes identified were: strengthening the public supply chain system, improving the private sector supply chain and devising a long-term strategy.

Reviewing responses in the strengthening public supply chain system, respondents emphasized improving the public supply chain system (EPSA) and advised the agency to consider supplying to private/NGO health facilities/retail outlets in addition to the public sector. A facility manager in a non-governmental faith-based organization health center elucidated his recommendation;

“We are providing service for the community with a fair price, this means sharing the burden of the public facilities. We are not making a profit from our service, and it’s well known. Note that, I don’t want to blame them since we are benefiting a lot. But our stand is to be prioritized equally with the public facilities. The prioritization could be due to the agency’s capacity to address all the needs. Hence, the government has to support the agency to ensure commodity security for maternal health medicines and other health products in general.” (Male facility head, Three years of experience).

Devising a long-term strategy to improve access was the second theme. Coded responses showed that formulating a strategy to build a strong local manufacturing and designing system for improved inventory management were the most important considerations of respondents. A respondent in a public hospital explained:

“It’s no doubt the local manufacturers support in all efforts to improve access lifesaving medicines. We all know during the covid pandemic, the local manufacturers are the ones who saved the community. They produced face masks, sanitizers and other personal protective supplies while the foreign vendors supply was disrupted. Local products also have a logistics and inventory management advantages, we shouldn’t wait months until the shipment arrives by sea or pay costly cargo.” (Male drug and supply manager, six years of experience).

In the improving the private sector theme, participants believed supporting the private pharmaceuticals suppliers could improve the overall access to maternal health medicines. Ethiopia faced a severe shortage of foreign currency to import basic goods and services in the last couple of years. To manage the shortage, the national bank of Ethiopia prioritized pharmaceuticals, input for manufacturing of edible oil and liquified petroleum gas as a first priority. However, the forex allocated for pharmaceuticals is consumed by the Ethiopian pharmaceutical supply agency while the private importers are underserved. While working on the long-term interventions, respondents recommended prioritizing resource allocation (including Forex) for MH medicine procurement both in public and private sectors.

## Discussion

Availability of maternal health medicines fell short of the 80% target set by WHO but was comparable to many other similar surveys. A study conducted in Uganda reported 36% average availability though it included contraceptives and child health commodities [17]. Another study conducted in Myanmar indicated that the overall availability of essential life-saving maternal and reproductive health (RH) medicines was 52.9% [34]. However, availability of those medicines makes the difference between life and death, and has the biggest impact on reducing maternal mortality [25]. Hence, priority attention to ensure commodity security should be given in countries like Ethiopia where the maternal mortality ratio (MMR) remains high. Unless access is ensured, the SDG target to reduce maternal mortality with no country having MMR over 140 per 100,000 live births by 2030 won’t be achieved [3].

Disparity in availability was observed across sectors, private pharmacies had the lowest availability of MHCs (40%) while the availability in public and mission sector was 72% each. A similar variation was reported in a study conducted in Uganda. It was found that the availability of commodities was highest in the mission sector (40%), followed by the public sector (38%), and was lowest in the private sector (31%) [17]. The lowest availability of MHCs in private pharmacies can be explained by the fact that these medicines are used in hospitals or clinical settings. Unless they are stockout in the public sector, there is no demand in the private pharmacies.

Availability of maternal health medicines like oxytocin and magnesium sulphate were high in the mission (100%) and the public facilities (89%) while private pharmacies rarely stocked oxytocin. Correspondingly, other studies reported very low oxytocin availability in private pharmacies (8.3%), while higher figures were observed in the public (73.3%) and faith-based health facilities (82.4%) [35, 36]. A review conducted in 12 countries in sub-Saharan Africa also reported, on average 19%, and 46% of facilities were stockout for oxytocin, and magnesium Sulphate respectively [37]. Even though it hits the WHO 80% index, health facilities, especially providing delivery service, are expected to stock both oxytocin and magnesium sulphate all the time since post-partum hemorrhage and eclampsia are major contributors of MMR in Ethiopia [38]. In addition, the availability of oxytocin declined while magnesium sulphate improved over the past five years in Addis Ababa [21].

Misoprostol 200mcg tablet and calcium gluconate were found in only 6%, and 30% of facilities visited for the study. The Uganda study also reported low availability of these medicines; calcium gluconate 28% and misoprostol 63% [17]. Similarly, a review in 12 countries in sub-Saharan Africa showed, on average, 73% of facilities were stockout for misoprostol [37]. Availability of misoprostol is a chronic hurdle and remained the same since 2016 in Addis Ababa [21]. The availability of alternative oxytocin for misoprostol or availability in kit form (misoprostol + mifepristone), and low prevalence of magnesium toxicity which requires calcium gluconate treatment could be a potential reason for not stocking.

This assessment found a relatively high availability of most of the antibiotics used for the treatment of STIs and other bacterial infections. Among antibiotics, the lowest availability in the public sector was for cefixime (33%), while it was benzathine penicillin in the private sector. Similarly, better availability of antibiotics was reported in Uganda with the lowest availability in the public sector for gentamicin (30%) [17]. Another study conducted in Addis Ababa on availability of commonly prescribed antibiotics reported the availability of 13 commonly prescribed antibiotics was high, on average 92.3% and 98.5% in the private and public pharmacies, respectively [20].

In the public sector, all MHCs were free to the patient; affordability was not an issue, with similarity to results found in Uganda [17]. In another study looking at the economic burden on patients for RMNCH services, 63% of 43 responding countries stated that women were exempt from paying for RMNCH medicines. However, commodities with wider indications than just RMNCH (i.e., antibiotics and steroids) were not provided free of charge as often [15]. As part of improving facility delivery to improve MMR and infant mortality rate, offering free maternal health services plays a pivotal role since payment was discouraging to seek health services [3].

The affordability of MHCs was a question in the rest of the three sectors in our study. Private hospital pharmacies were the most expensive, 87% of the medicines were found unaffordable; private pharmacies followed with 83% and the NGO/mission sector with 71%. Other studies conducted in Ethiopia also reported less affordability in the private sector compared to the public and NGO/mission sector since medicine prices are not regulated and there is no agreed-upon mechanism to determine the final/patient level price of drugs [10, 20, 39]. However, better affordability was reported in Uganda, 74% of SRHCs were considered affordable in the mission sector, and 64% of those in the private sector [17].

The average availability of MHCs that could be sourced locally was higher than imported medicines. Likewise, the high availability of locally produced medicine in Ethiopia was reported by WHO in 2016. The WHO assessment aimed to compare price and availability of locally produced and imported medicines in Ethiopia and identified that, locally produced medicines had greater mean availability (48%) than imported products in all sectors ( public sector 48% vs 19%, private sector 54% vs 35%, other sectors 55% vs 32%) [40]. Hence, policymakers should use additional and effective policy instruments like establishing robust regulatory infrastructures, introducing tax holidays, tax exemptions, bank loans with discounted interest rates, etc. to boost local pharmaceutical manufacturing capacity [20].

This study showed medicines with a higher number of registered suppliers appear to have higher percent availability in the city. A cross-country analysis in 75 Countdown to 2015 countries, including Ethiopia, from eight regions to identify problems with specific commodities and determinants of access on eight policy and system indicators for each of the 15 tracer commodities was published in 2018. The analysis showed registration of commodities was one of the most challenging factors [15]. EFDA has to improve the identified internal inefficiencies in order to improve clients satisfaction to increase the number of registered suppliers [41].

Respondents in this study claimed that key challenges to access maternal health medicines in their facility were frequent stockouts, requested medicines were not supplied and the cost of medicines was high in the private sector. Similarly, a survey conducted in Dessie reported the reason for stock-outs of priority lifesaving MCH medicines was the public supply agency (EPSA) did not supply adequate products [42]. Correspondingly, frequent stock-outs, issues or delays with the supply of the commodities at the public facility and costs to patients in the private and mission sectors were major barriers in Uganda [17]. It is not difficult to imagine the situation in remote areas and regional towns would look like if access to priority lifesaving MHCs in the capital, is hampered by multiple challenges.

## Conclusion

Availability of priority MHCs in Addis Ababa was fairly high according to the WHO availability index, even though it fell short of the target. Besides the availability, MHCs were free of charge in the public sector while they were not affordable in the other sectors. Key challenges to access maternal health medicines were frequent stockouts, requested medicines were not supplied and the high cost of medicines. All relevant actors including ministry of health, EPSA, FDA, policymakers and implementing partners in the supply of maternal health medicines has to act collaboratively to tackle the inadequate supply and high cost of priority maternal health medicines in order to hit the target to reduce maternal mortality by 2030,

### Limitation of the study

The study was conducted in a single survey area focused on maternal health medicines though the survey could be national and might had to consider other essential medicines. Future studies could consider multiple survey areas with wider geographic coverage.

## Supplementary Information

**Additional file 1. **Median Price of maternal health medicines and Daily Wags Required for a full dose/ monthly treatment, in Addis Ababa, January 2021.

## Data Availability

The datasets used and/or analysed during the current study are available from the corresponding author on reasonable request.

## Abbreviations

MHC: Maternal Health Commodity
HAI: Health action international
WHO: World health organization
LPGW: Lowest-paid government worker
EPSA: Ethiopian pharmaceuticals supply agency
