# Supplementary material for: Availability and affordability of priority lifesaving maternal health medicines in Addis Ababa, Ethiopia
Source: BMC Health Serv Res. 2022 Apr 20;22:524. doi: 10.1186/s12913-022-07793-x (PMC9019981; doi:10.1186/s12913-022-07793-x)
Supplement: Supplementary file 1 — Additional file 1. Median Price of maternal health medicines and Daily Wags Required for a full dose/ monthly treatment, in Addis Ababa, January 2021. [file 12913_2022_7793_MOESM1_ESM.docx]

Median Price of maternal health medicines and Daily Wags Required for a full dose/ monthly treatment, in Addis Ababa, January 2021

| MH Medicine | Median Price | | | | Full dose | Total Price | | | | Days of wage | | | | |
| --- | --- | --- | --- | --- | --- | --- | --- | --- | --- | --- | --- | --- | --- | --- |
|  | Private Pharmacy | Private Hospital | NGO/ Mission | Public |  | Private Pharmacy | Private Hospital | NGO/ Mission | Public | Private Pharmacy | Private Hospital | NGO/ Mission | Public |  |
| Ampicillin 1g Injection | 25 | 41 | 14.4 | 0 | 20 | 500 | 820 | 288.1 | 0 | 13.6 | 22.4 | 7.9 | 0 |  |
| Azithromycin 500mg Capsule | 12.8 | 49 | 15 | 0 | 1 | 12.8 | 49 | 15 | 0 | 0.3 | 1.3 | 0.4 | 0 |  |
| Benzathine benzylpenicillin 2.4 million units in vial Injection | NA | NA | 38.5 | 0 | 1 | NA | NA | 38.475 | 0 | NA | NA | 1.0 | 0 |  |
| Calcium-gluconate 10% in 10ml Injection | NA | 155 | 26 | 0 | 1 | NA | 155 | 26 | 0 | NA | 4.2 | 0.7 | 0 |  |
| Cefixime 400mg Tablet | 42.2 | 72.8 | 25 | 0 | 1 | 42.2 | 72.8 | 25 | 0 | 1.2 | 2.0 | 0.7 | 0 |  |
| Dexamethasone 4mg/ml in 1ml Injection | 15 | 18 | 15 | 0 | 8 | 120 | 144 | 120 | 0 | 3.3 | 3.9 | 3.3 | 0 |  |
| Gentamicin 40mg/ml in 2 ml Injection | 15 | 2 | 31 | 0 | 10 | 150 | 20 | 310 | 0 | 4.1 | 0.5 | 8.5 | 0 |  |
| Hydralazine 20mg Injection | NA | 140 | 72 | 0 | 1 | NA | 140 | 72 | 0 | NA | 3.8 | 2.0 | 0 |  |
| Magnesium sulfate 50% in 10ml Injection | 140 | 75 | 30 | 0 | 18 | 2520 | 1350 | 540 | 0 | 68.7 | 36.8 | 14.7 | 0 |  |
| Methyldopa 250mg Tablet | 4 | 1 | 3 | 0 | 90 | 360 | 90 | 270 | 0 | 9.8 | 2.5 | 7.4 | 0 |  |
| Metronidazole 500mg in 100 ml vial Infusion | 25 | 29.85 | 17.4 | 0 | 30 | 750 | 895.5 | 520.5 | 0 | 20.5 | 24.4 | 14.2 | 0 |  |
| Mifepristone + Misoprostol 200mg + 200mcg Tablet | 148 | 131 | 120 | 0 | 1 | 148 | 131 | 120 | 0 | 4.0 | 3.6 | 3.3 | 0 |  |
| Misoprostol 200 mcg Tablet | NA | NA | NA | 0 | 4 | NA | NA | NA | 0 | NA | NA | NA | 0 |  |
| Nifedipine (Immediate release) 20mg Capsule | 1 | 0.85 | 0.9 | 0 | 8 | 8 | 6.8 | 7.36 | 0 | 0.2 | 0.2 | 0.2 | 0 |  |
| Oxytocin 10IU Injection | NA | 46.25 | 40 | 0 | 2 | NA | 92.5 | 80 | 0 | NA | 2.5 | 2.2 | 0 |  |
| Ringer lactate Lactated ringer 1L Infusion | 60 | 90 | 56 | 0 | 3 | 180 | 270 | 168 | 0 | 4.9 | 7.4 | 4.6 | 0 |  |
| Sodium chloride 0.9 % Isotonic in 1L Infusion | 60 | 68 | 42.5 | 0 | 3 | 180 | 204 | 127.5 | 0 | 4.9 | 5.6 | 3.5 | 0 |  |
| TD (Tetanus, Diphtheria Toxoid) or TT (Tetanus Toxoid) | NA | 95 | 42.2 | 0 | 4 | NA | 380 | 168.8 | 0 | NA | 10.4 | 4.6 | 0 |  |
